# Two Epitope Regions Revealed in the Complex of IL-17A and Anti-IL-17A V_H_H Domain

**DOI:** 10.3390/ijms232314904

**Published:** 2022-11-28

**Authors:** Olga Kostareva, Arina Svoeglazova, Ilya Kolyadenko, Alexey Nikulin, Stanislav Evdokimov, Uliana Dzhus, Azat Gabdulkhakov, Svetlana Tishchenko

**Affiliations:** 1 Institute of Protein Research, Russian Academy of Sciences, Institutskaya, 4, 142290 Pushchino, Russia; 2 CJSC Biocad, ul.Svyazi., 34-A, sett. Strelna, 198515 Saint-Petersburg, Russia

**Keywords:** interleukin 17A in complex with V_H_H domain, netakimab, crystal structure

## Abstract

Interleukin-17 (IL-17) is a cytokine produced by the Th17 cells. It is involved in chronic inflammation in patients with autoimmune diseases, such as rheumatoid arthritis, systemic lupus erythematosus, multiple sclerosis, and psoriasis. The antibodies targeting IL-17 and/or IL-17R are therapy tools for these diseases. Netakimab is an IL-17A-specific antibody containing a *Lama glama* V_H_H derivative domain and a VL variable domain. We have determined the crystal structure of the IL-17A-specific V_H_H domain in complex with IL-17A at 2.85 Å resolution. Certain amino acid residues of the three complementary-determining regions of the V_H_H domain form a network of solvent-inaccessible hydrogen bonds with two epitope regions of IL-17A. The β-turn of IL-17A, which forms the so-called epitope-1, appears to be the main region of IL-17A interaction with the antibody. Contacts formed by the IL-17A mobile C-terminal region residues (epitope-2) further stabilize the antibody–antigen complex.

## 1. Introduction

The interleukin-17 (IL-17) family consists of homo- or heterodimeric cytokines designated as IL17A-F [1]. IL-17A is primarily produced by the Th17 cells evolved from activated CD4^+^ T helper cells [2], but there are other cell types also secreting this cytokine: CD8^+^ T cells [3], γδ T cells [4,5]), invariant natural killer T (iNKT) cells [6], natural killer (NK) cells [7], innate lymphoid cells (ILCs), lymphoid-tissue inducer (LTi)-like cells [8], type 3 innate lymphoid cells (ILC3) [9], neutrophils [10], Paneth epithelial cells [11], and mast cells [12,13].

Acting in synergy with other cytokines (such as TNFα, IFNγ, or IL-22), IL-17A directly activates keratinocytes and dermal fibroblasts to produce cytokines (e.g., IL-6, TNFα, IL-1β, IL-20 family cytokines, GM-CSF), chemokines (CXCL1, CXCL2, CCL20, CXCL8/IL-8), and anti-microbial peptides [14].

IL-17A signaling occurs through its membrane-bound receptors, IL-17RA and IL-17RC [15,16] and elicits multiple inflammatory responses and immune disorders, such as psoriasis, psoriatic arthritis, rheumatoid arthritis, and multiple sclerosis [17]. IL-17A can also signal through a complex of IL-17RA and IL-17RD, although additional research is required to understand the role and functional consequences of this receptor complex in IL-17A signaling [18]. IL-17A binds to IL-17RA with nanomolar affinity, and the spatial structure of the complex is available [19]. The antibodies block the interaction of IL-17A with receptor by binding to one of the partners. Thus, IL-17A-specific antibodies prevent the binding of IL-17A to its receptors by competing for a receptor-binding site.

Therapies based on blocking the IL-17 signaling pathway by inhibitors are effective treatments for patients with autoimmune diseases and inflammation. Monoclonal antibodies ixekinumab, secukinumab, and netakimab target IL-17A, while brodalumab targets the receptor, IL-17RA. These antibodies are widely used in the treatment of psoriasis, psoriatic arthritis, and ankylosing spondylitis [20]. Secukinumab and ixekinumab are recombinant high-affinity human monoclonal antibodies belonging to the IgG1/kappa isotype [21,22,23]. Netakimab was designed by the Russian biotechnological company BIOCAD and registered in the Russian Federation as «Efleira»; it is successfully used for the treatment of moderate to severe plaque psoriasis [24,25]. The light chain of the monoclonal antibody netakimab belongs to the VK3 structural family and contains domains Cl and VL. The heavy chain consists of three constant domains (CH3, CH2, CH1); the variable domain VH is substituted with a variable domain of a non-canonical antibody—IL-17A-specific V_H_H domain. Non-canonical antibodies containing only a heavy chain with extended hypervariable Complementary-Determining Regions (CDRs) are usually found in some species of the Camelidae family. Recently, we determined the crystal structure of the netakimab Fab fragments [26].

In this article, we present the first crystal structure of IL-17A bound to the IL-17A-specific V_H_H domain determined at 2.85 Å resolution. The V_H_H domain binding site of IL-17A overlaps with the site of interaction with the IL-17RA. Thus, netakimab which is based on the V_H_H domain, prevents IL-17A binding to its receptor by competing for a receptor-binding site.

## 2. Results

### 2.1. Structure of the V_H_H Domain in Complex with IL-17A

Upon designing the netakimab, several V_H_H domain variants were obtained. One of them, anti-IL-17A-76, having a point replacement Tyr76Asn. It is known that mutations on the surface of a protein often affect its crystallizability. Neither the wild-type domain nor the mutants, but the Tyr76Asn, formed suitable crystals of the IL-17A/anti-IL-17A complex. Thus, choice of such a mutant form of the anti-IL-17A is determined by its ability to form a stable complex with IL-17A and form crystals suitable for X-ray structural analysis. Affinity of the anti-IL-17A-76 is quite similar to that of the anti-IL-17A (V_H_H domain of netakimab antibody) (Table 1). The structure of IL-17A/anti-IL-17A-76 was refined to 2.85 Å resolution. The asymmetric unit contains IL-17A monomer (molecule A or A*) binding to anti-IL-17A-76 molecule.

**Figure 1:**
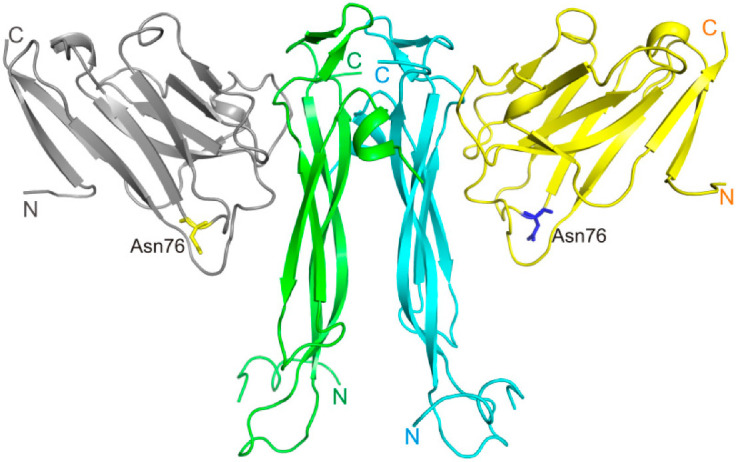
The structure of IL-17A/anti-IL-17A-76 complex (PDB code 8B7W). IL-17A monomers are shown in blue and green, anti-IL-17A-76—in yellow and grey.

The IL-17A dimer sandwiched between two anti-IL-17A-76 forms two IL-17A/anti-IL-17A-76 interaction sites (Figure 1).

**Figure 2:**
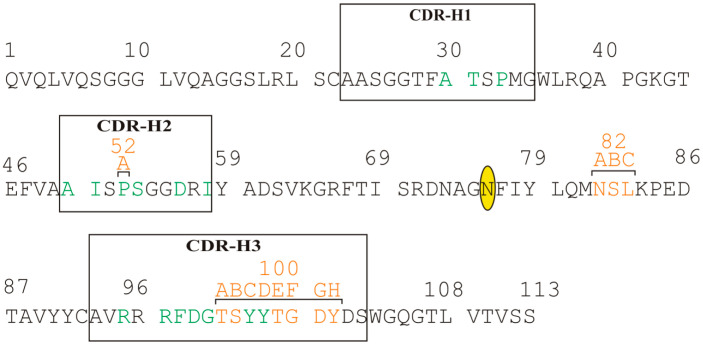
Amino acid sequence of anti-IL-17A-76 (the numbering of amino acids is presented in accordance with the Kabat Database). The CDR regions are shown with frames. Amino acids involved in the interaction with IL-17A are shown in green. Point mutation Y76N is shown by ellipse.

The IL-17A/anti-IL-17A-76 contact surface area is 960 Å^2^: most of the surface (840 Å^2^) includes contacts with molecule A of IL-17A: 8 hydrogen-bonds (H-bonds) and 14 hydrophobic contacts; molecule A* forms only one H-bond and two hydrophobic contacts with anti-IL-17A-76 (120 Å^2^). The main contributors into the anti-IL-17A-76 with the cytokine interaction are CDR-H2 (3 H-bonds, 4 hydrophobic contacts) and CDR-H3 (5 H-bonds and 5 hydrophobic contacts) regions (Figure 2). The list of contacts in IL-17A/anti-IL-17A-76 complex is given in Table S1.

### 2.2. Modeling of the IL-17A/Netakimab Fab Fragment Complex

**Figure 3:**
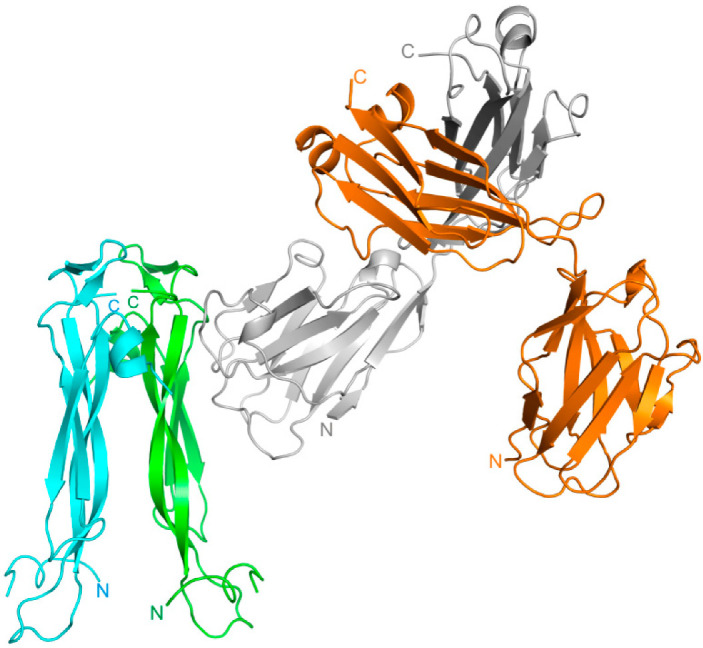
Superposition of the netakimab Fab fragment structure (PDB code 6QKD) on the structure of the V_H_H domain in complex with IL-17A (PDB code 8B7W). The heavy chain is shown in grey, the light chain is in orange. IL-17A monomers are shown in blue and green.

Recently we solved the structure of the netakimab Fab fragment containing V_H_H domain (PDB code 6QKD). The molecule has a V-shaped conformation [26], and it differs from the classical Fab fragments, which is determined by the specificity of the V_H_H domain CDR-H3 loop sequence. The superposition of the V_H_H domain in complex with IL-17A and the netakimab Fab fragment structures showed that the light chains do not interact with the cytokine (Figure 3).

The kinetic analysis of the Fab fragment and V_H_H domain interactions with the IL-17A indicated that the IL-17A/Fab fragment complex is formed an order of magnitude slower than IL-17A/V_H_H (Table 2), but the stability of the complexes is almost the same [26]. The increasing rate of association of V_H_H domain with the IL-17A is due to the small size of the V_H_H domain compared with the Fab fragment.

### 2.3. The Structure of Anti-IL-17A-N Mutant Form

**Figure 4:**
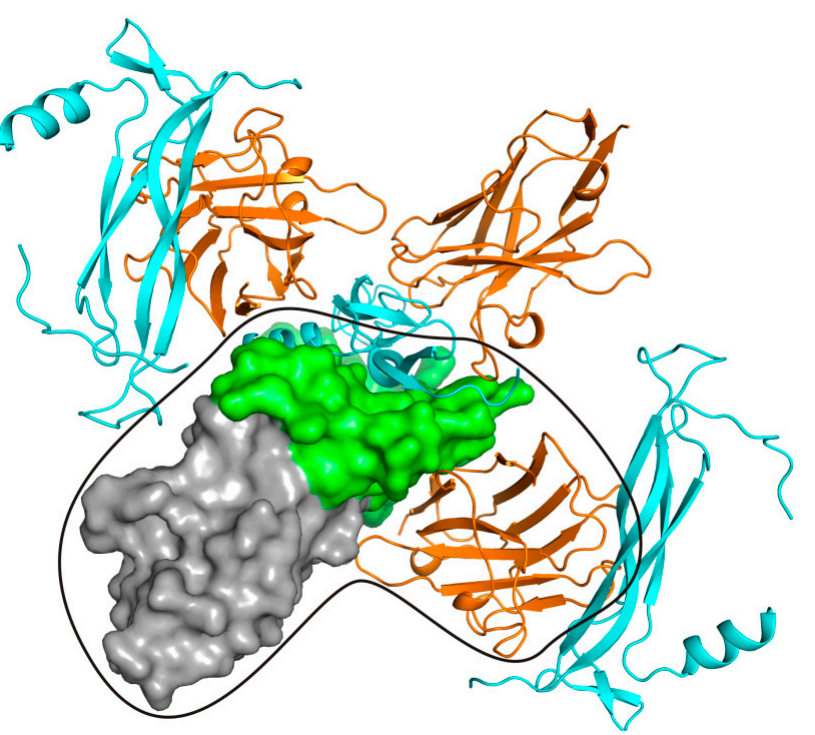
The surfaces show the IL-17A monomer (green) associated with the anti-IL-17A-76 (grey) in the asymmetric part of the cell. Ribbon models show the anti-IL-17A-76 (orange) and the second IL-17A monomer (blue). The IL-17A monomer and the anti-IL-17A-76 in contact with it are outlined.

An analysis of the IL-17A/anti-IL-17A-76 molecules crystal packing (Figure 4) showed additional extensive crystal contact of the IL-17A monomer with the anti-IL-17A-76 located in the neighboring cell (Table S2). The crystal contact has a large area (580 Å^2^), comparable to the main contact (960 Å^2^).

**Figure 5:**
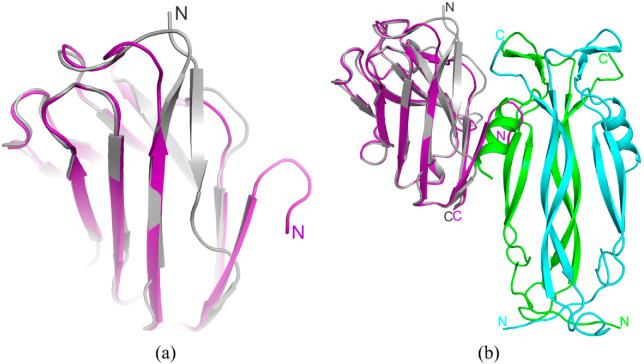
Comparison of anti-IL-17A-76 and anti-IL-17A-N mutant form structures in the free state and in the complex with IL-17A. (**a**) Superposition of the anti-IL-17A-76 (grey) structure from the IL-17A/anti-IL-17A-76 complex and the structure of the anti-IL-17A-N mutant form—purple (PDB code 6RBB). (**b**) Superposition of the anti-IL-17A-N (purple) structure (PDB code 6RBB) on the structure of the anti-IL-17A-76 (grey) in the complex with IL-17A. IL-17A monomers are shown in blue and green.

Based on the analysis of the anti-IL-17A-76 in complex with IL-17A structure, we suggested that removal of the N-terminal residues from 7 to 10 positions (SGGG) (Table 3) would lead to closeness of polar Gln6 with the hydrophobic region (Lue18, Leu20, and Val109) and probable repulsion. On the other hand, this repulsion could lead to a shift toward C-terminal β-strand (Glu105-Ser112), forming contacts (Figure 5a). Thereby, it could create steric obstacles for additional contacts of anti-IL-17A-76 with IL-17A (Figure 4). A mutant form with substitutions in the N-terminal region (anti-IL-17A-N, Table 3) was obtained and crystallized, and its structure was determined. At the result, the N-terminal part of the anti-IL-17A-N formed a β-strand, which was moved away from the V_H_H domain “body” due to electrostatic repulsion (Figure 5a).

Kinetic analysis of the IL-17A interactions with the anti-IL-17A-76 and with the anti-IL-17A-N showed that the mutant domain affinity did not change. The association and dissociation rate constants are almost the same (Table 4).

## 3. Discussion

### 3.1. Interactions of the V_H_H Domain with IL-17A

**Figure 6:**
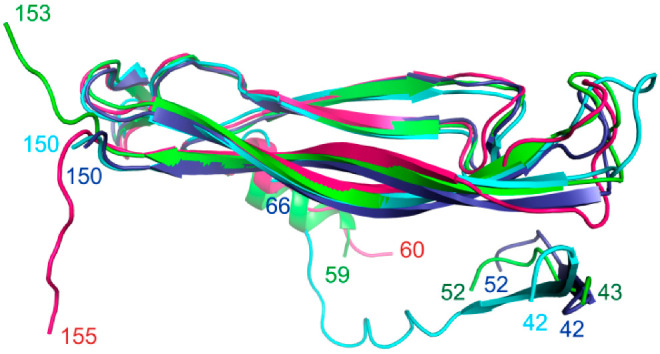
The mobility of structural elements of IL-17A in a free state and in complexes with ligands. Superposition of the structures: IL-17A (PDB code 4HR9) is dark blue, IL-17A/IL-17RA (PDB code 4HSA)—blue, IL-17A/secukinumab Fab fragment (PDB code 6WIO)—red, and IL-17A/anti-IL-17A-76 (PDB code 8B7W)—green.

The IL-17A monomer molecule has a structured (two antiparallel β-sheets) and unstructured regions that are not seen in the crystal structure. The interaction of IL-17A with the receptor or an antibody arranged the unstructured regions of the cytokine located at the N- and C-termini (Figure 6). The IL-17RA binds to an extended area of IL-17A and arranges approximately 20 amino acid residues of the N-terminal region. The classical antibody secukinumab interacting with the cytokine arranges the IL-17A C-terminal region. In the IL-17A/anti-IL-17A-76 complex, the region 58–69 aa of IL-17A becomes ordered into a helix and the IL-17A C-terminal becomes structured (Figure 6).

**Figure 7:**
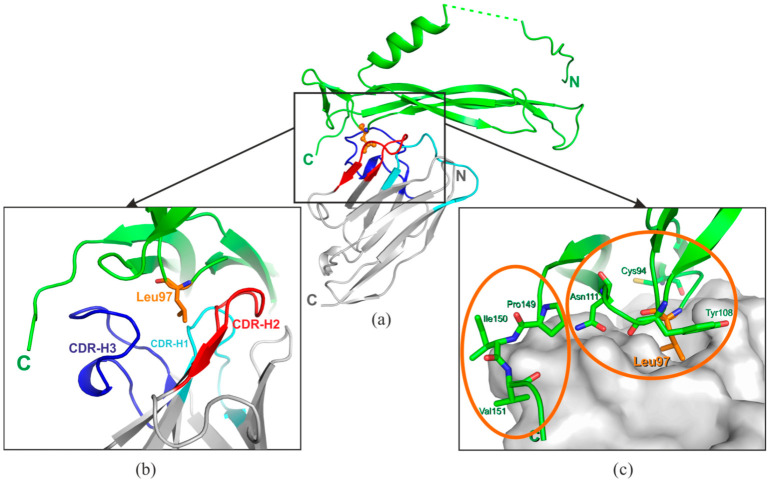
Detailed analysis of the epitope-paratope site in the IL-17A/anti-IL-17A-76 complex. IL-17A—green, anti-IL-17A-76—grey. CDR-H1—blue, CDR-H2—red, CDR-H3—dark blue. (**a**) A general view of the IL-17A monomer/anti-IL-17A-76 structure. (**b**) A general view of the complex interface (ribbon model). (**c**) Amino acid residues of IL-17A involved in the interaction with anti-IL-17A-76. Ellipses enclose two areas of the epitope.

The anti-IL-17A-76 paratope region comprises a hydrophobic pocket formed by the three CDRs (Figure 7). The IL-17A epitope could be divided into 2 regions: the epitope-1 located in the structured β-fold, and the epitope-2 in the flexible C-terminal region. The Cys94, Leu97, Asn111 of the epitope-1 and Ile150, Val151 of the epitope-2 form solvent-inaccessible H-bonds with the anti-IL-17A-76 CDRs residues (Table S1).

The Leu97 located in the IL-17A beta-turn adopts a sterically favorable conformation for the interaction with the anti-IL-17A-76 hydrophobic pocket (Figure 7). The Leu97 plays the role of a “knob” fixing the anti-IL-17A-76 CDRs residues in the cavity. The Tyr108, Asn111 of IL-17A and the residues of the CDR-H1 (Pro33), CDR-H2 (Ile58, Ser52, Asp56), and CDR-H3 (Arg95, Ser100B) form “walls” of the paratope/epitope-1 pocket. The mobile C-terminal part of IL-17A (the epitope-2) interacts with the anti-IL-17A-76 CDR-H3 residues (Table S1).

One may assume that the epitope located in the beta-turn plays the main role in the specific interaction with the anti-IL-17A-76, and contacts formed by the mobile C-terminal region contacts fix the position of the antibody on IL-17A.

**Figure 8:**
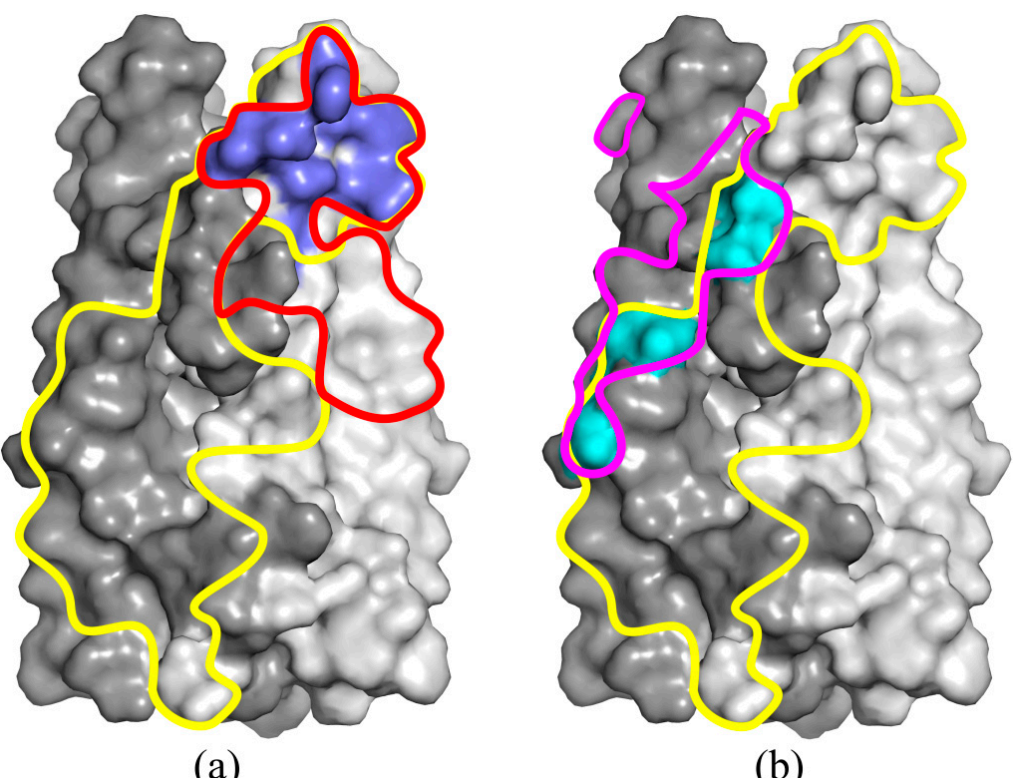
Surfaces of IL-17A monomers are shown in shades of grey. The yellow outline shows contacts with the IL-17RA receptor. (**a**) The main contacts of the anti-IL-17A-76 with IL-17A are shown in a red outline. The dark blue color shows the intersection between the anti-IL-17A-76 and IL-17RA contacts with IL-17A. (**b**) The crystal contact region is depicted with a crimson outline. The contact intersections area is shown in cyan.

The binding sites of the anti-IL-17A-76 and the IL-17RA on the IL-17A overlap (Figure 8a). The Asn111 of the epitope-1 and Ile150 of the epitope-2 form solvent-inaccessible H-bonds both in IL-17A/anti-IL-17A-76 and IL-17A/IL-17RA complexes (Table S1). The solvent-inaccessible H-bonds play a crucial role in specific intermolecular interactions [27]. Thus, contacts of the conserved residues Asn111 and Ile150 of IL-17A with netakimab (based on anti-IL-17A) may play a decisive role in the competition between the antibody and the receptor for the cytokine binding.

### 3.2. Crystal Contacts between of the V_H_H Domain and IL-17A Are of No Functional Significance

The crystal packing of the IL-17A/anti-IL-17A-76 demonstrated the additional contacts of the V_H_H domain located in the neighboring asymmetric unit with IL-17A monomer. The additional contact has a large area (~580 Å^2^) comparable to the main contact area of ~960 Å^2^, and it intersects with the contacts of IL-17A with IL-17RA (PDB code 4HSA) [28] (Figure 8).

We suggest that the mutant form of the anti-IL-17A domain with substitutions in the N-terminal region (anti-IL-17A-N) will cause a steric hindrance to the formation of crystal contacts with IL-17A.

Indeed, the superposition of the anti-IL-17A-N and anti-IL-17A-76 in the complex with IL-17A structures (Figure 5b) demonstrated that the changes in the N-terminus do not allow the formation of crystal contacts. Kinetic analysis of the IL-17A/anti-IL-17A and anti-IL-17A/anti-IL-17A-N interactions showed that their affinities to the cytokine are virtually the same (Table 4).

Thus, despite the plethora of the crystal contacts in the IL-17A/anti-IL-17A-76 complex and their intersection with receptor contacts, they have no functional significance.

### 3.3. Comparative Structural Analysis of the IL-17A Epitope Regions

At the present time, several structures of the IL-17A complexes with antibodies having a proven inhibitory effect are available (Table 5). The affinities of these antibodies and the anti-IL-17A for IL-17A are comparably high. We carried out a detailed comparative structural analysis of the epitope regions in the IL-17A/anti-IL-17A-76 interactions and the complexes of the IL-17A with functionally significant antibodies.

At first, we compared the complexes of IL-17A with antibodies (secukinumab and CAT-2200) for which binding regions overlap with the anti-IL-17A-76 site. The Table 5 shows affinity for each antibody/IL-17A complex and half-maximal inhibitory concentrations.

#### 3.3.1. IL-17A/Secukinumab Fab Fragment Complex

The affinity of the secukinumab [30] for IL-17A is comparable that of netakimab V_H_H domain (Table 5). The contact surface areas in the complexes of IL-17A with secukinumab Fab fragment (PDB code 6WIO) and anti-IL-17A-76 are almost the same; the epitopes partially overlap (Figure S1a) and include 10 IL-17A residues (Table S1, Figure S2).

Nevertheless, participation of IL-17A monomers in binding to anti-IL-17A-76 and secukinumab Fab fragment is different. Most (approximately 90%) of the anti-IL-17A-76 interactions occur on one of the IL-17A monomers, while in the complex of IL-17A with secukinumab, both IL-17A monomers almost equally contribute to binding (Table S1). Most of the IL-17A/secukinumab interactions are formed by the heavy chain residues (CDR-H2 and CDR-H3). The number of the hydrophobic contacts in IL-17A/anti-IL-17A-76 complex is similar to that of IL-17A/secukinumab, but secukinumab forms twice as many H-bonds with IL-17A than anti-IL-17A-76. Nonetheless, the number of solvent-inaccessible H-bonds in both complexes is almost equal (Table S1).

The epitope region recognized by secukinumab forms the solvent-inaccessible H-bonds with paratope residues. This region can be divided into two parts as well as in the IL-17A/anti-IL-17A-76 complex. The secukinumab-binding epitope-1 is located on the IL-17A β-turn near the epitope-1 for anti-IL-17A-76. The secukinumab-binding epitope-2 is in the IL-17A C-terminal part (Figure S1b, Table S1) and matches the epitope-2 in IL-17A/anti-IL-17A-76 complex.

The epitope regions in IL-17A/secukinumab complex resemble two bulges containing Asn111 (the epitope-1) and Pro149 (the epitope-2). We assume that the epitope-2 has a leading role in the antigen–antibody interactions.

#### 3.3.2. IL-17A/CAT-2200 Fab Fragment Complex

The affinity of the CAT-2200 antibody Fab fragment [31] for IL-17A is 20 fold lower compared to the affinities of the secukinumab Fab fragment and the netakimab V_H_H domain (Table 5). Epitopes in the IL-17A/CAT-2200 and IL-17A/anti-IL-17A-76 complexes partially overlap (Figure S3a).

The contact region includes 14 hydrophobic interactions and 8 hydrogen bonds. Two H-bonds are inaccessible to the solvent (Table S1). The two epitope regions for CAT-2200 are located on different IL-17A monomers. Both chains of the CAT-2200 are involved in binding to the cytokine (Figure S3a).

The CAT-2200 binding epitope-1 is formed by residues located in a loop forming the turn α-helices (Figure S3B) that become organized into a full helix in the complex with anti-IL-17A-76 (Figure 6). The CAT-2200 binding epitope-2 is located in the IL-17A β-hairpin region (Figure S3c).

The reduced affinity of the CAT-2200 Fab fragment for the IL-17A can be explained by the peculiarity of the epitope relief in this complex (the bulges are absent).

Fab fragments of the antibody 6785 (PDB code 4QHU), the computationally designed functional antibody h142 (PDB code 5N7W), and the HB0017 (PDB code 7WKX), recognize a IL-17A region, which is different from that of the anti-IL-17A-76. However, the data on the affinity of the Fab 6785 for IL-17A and IC_50_ are missing; the affinity of the Fab fragment h142 for the cytokine is reduced as compared to that of other tested antibodies [33]. Since the HB0017 has the highest affinity for IL-17A among the known IL-17A-specific antibodies [32] (Table 5), we carried out a comparative structural analysis of the interactions of IL-17A with the HB0017 Fab fragment and the anti-IL-17A-76.

#### 3.3.3. Comparative Analysis of the Epitope-Paratope Regions in the IL-17A/HB0017 Fab Fragment and IL-17A/Anti-IL-17A-76 Complexes

The IL-17A/HB0017 contact surface area (802 Å^2^) is comparable to that of the IL-17A/IL-17A-76 and the IL-17A/secukinumab (Table S1). The main contact of HB0017 on IL-17A is located in a single node formed by two IL-17A monomers—the β-turn of IL-17A. The heavy (Figure S4b) and light (Figure S4c) chains of the HB0017 take part in the formation of the epitope–paratope interactions. Two solvent inaccessible H-bonds (Table S1) formed by side chains of Pro 82 and Glu 83 with HB0017 appear to play a key role in the antibody–antigen interactions.

## 4. Materials and Methods

### 4.1. Production and Purification of VHH Domain of Netakimab Antibody

The pET28a vector carrying V_H_H domain gene of netakimab antibody (anti-IL-17A) was used for transformation in *E. coli* BL21(DE3)/Rosetta. The production and purification of anti-IL-17A were carried out according to a previously published method [26]. Preparations were analyzed by gel electrophoresis under denaturing conditions at every stage.

### 4.2. Production and Purification of Mutant Forms of Anti-IL-17A

The mutant form of anti-IL-17A with point replacement Tyr 76 Asn—anti-IL-17A-76 (Table 1)—was obtained by quick change PCR method using primers For, 5′- TCCAGAGACAACGCCGGATACTTTATTTATCTGCAAATGAAC-3′ and Rev, 5′-GTTCATTTGCAGATAAATAAAGTATCCGGCGTTGTCTCTGGA-3′ and pET-28a/anti-IL-17A plasmid as a template.

The mutant form of anti-IL-17A with modified N-terminal sequence—anti-IL-17A-N (Table 3)—was PCR-amplified using primers for, 5′-GAAGTTCAACTGGTGCAGCAGGCTGGGGGCTCT-3′ and Rev, 5′-AGAGCCCCCAGCCTGCTGCACCAGTTGAACTTC-3′ and pET-28a/anti-IL-17A plasmid as a template. The obtained genetic constructions were validated by sequencing.

The production and purification of anti-IL-17A mutant forms were carried out according to a protocol for anti-IL-17A. Preparations were analyzed by gel electrophoresis under denaturing conditions at every stage. Fractions containing the purified protein were collected and concentrated to 10 mg/mL.

### 4.3. Production and Purification of the IL-17A

The pAC28 vector carrying IL-17A gene without N-signal sequence and with double mutation (N68D/C129S) [26,28] was used for transformation in *E. coli* BL21(DE3)/Rosetta. The transformants were grown in LB medium in the presence of kanamycin (50 µg/mL) and chloramphenicol (10 μg/mL) at 310 K with a rotating speed of 180 rpm. The induction was initiated at the OD_600nm_ = 0.6–0.8 o.u. with IPTG (0.3 mM). The bacteria were harvested by centrifugation 3 h after induction.

The procedure of inclusion bodies isolation, denaturation, and following refolding of IL-17A was similar with the protocol of Meng [34]. The cell pellet was suspended in a buffer A containing 50 mM Tris-HCl, pH 8.0, 2 mM Na_2_EDTA, centrifuged at 15,000× *g* for 15 min. The cell pellet was resuspended in buffer A with the addition of 1 mM *PMSF* and beta-mercaptoethanol (5 mM). The cells were disrupted by sonication for 45 min at 277 K. The buffer B containing 20 mM Tris-HCl, pH 8.0, 300 mM NaCl, 2 mM Na_2_EDTA, 25% sacharose, 1.5%, Triton X-100, 1.5% sodium deoxycholate was added in equal volume to the cell lysate, mixed and centrifuged at 15,000× *g* for 10 min at 277 K. The obtained inclusion bodies pellet was resuspended in 1:1 ratio with buffers A and B and centrifuged at 15,000× *g* for 10 min at 277 K. The pellet was resuspended in buffer C containing 20 mM Tris-HCl 8.0, 100 mM NaCl and centrifuged at 15,000× *g* for 10 min at 277 K. The procedure repeated 2–3 times. Further, the inclusion bodies pellet was resuspended in a buffer D containing 50 mM Tris-HCl, pH 8.0, 6 M guanidine HCl, 10 mM DTT in a ratio of 20 mL buffer D per 1 g of pellet. The solution incubated with vigorous stirring at room temperature for 14 h, then it was diluted 20 times with a refolding buffer E containing 100 mM Tris-HCl, pH 8.0, 1 M L-Arginine, 0.3 mM GSH, 0.3 mM GSSG and incubated at 277 K with slow mixing (100 rpm) for 40–60 h. The refolded protein passed through a 0.45 mkm filter, precipitated with 3.2 M ammonium sulfate and incubated for 12–16 h at 277 K with medium stirring. The precipitated protein was harvested by centrifugation at 15,000× *g* for 15 min at 277 K. The pellet was suspended in buffer F (150 mM NaCl, 50 mM Sodium Acetate, pH 5.5) and purified by the size exclusion chromatography on the Superdex G-75 (GE Healthcare). Fractions containing the purified protein were collected and concentrated to 10 mg/mL.

### 4.4. Analysis of the Anti-IL-17A and Its Mutant Forms with IL-17A Interactions by Surface Plasmon Resonance Approach (SPR)

Measurements were carried out using OCTET RED96 apparatus (Pall Forte Bio) in a running buffer (PBS supplemented with 0.1% BSA and 0.1% Tween-20) at 303 K. Biosensors AR2G (Pall Forte Bio) were rehydrated for one hour in deionized water and activated in 20 mM1-Ethyl-3-(3-dimethylaminopropyl) carbodiimide hydrochloride (EDC)/10 mM N-hydroxy(sulfo) succinimide (Sulfo-NHS). IL-17A at a concentration of 25 μg/mL in 10 mM sodium acetate (pH 4.0) was nonspecifically (through the amino groups) immobilized on the biosensor surface. After the neutralization with 1 M Ethanolamine (pH 8.5), sensors were immersed into wells containing solutions of the anti-IL-17A or its mutant forms at concentrations ranging from 1.1 nM to 10 nM. Sensors were then immersed into the running buffer for the subsequent dissociation stage; duration of association stage was 1000 s, and the dissociation stage was 3600 s. The curves obtained were analyzed using the Octet System Data Analysis software and 1:1 Langmuir binding model. The measurements were repeated three times.

### 4.5. Preparation of IL-17A/Anti-IL-17A-76 Complex and Crystallization

The anti-IL-17A-76 and IL-17A were mixed at molar ratio of 2.3:1, incubated for 10 min at 297 K and applied to Superdex G-75 column preliminarily equilibrated with buffer F. The fractions containing complex were collected and concentrated to 10 mg/mL.

The crystals of IL-17A/anti-IL-17A-76 complex were obtained by the vapor-diffusion method using as precipitant solution No. 8 of Cryo Crystal Screen 2 (Hampton Research) containing 1.05 M NaCl, 7% *v*/*v* ethanol, 30% glycerol. The drops were made by mixing the complex with the well solution at a 1:1 (*v*/*v*) ratio at 297 K, and crystals grew to maximum dimensions of 40 µm × 30 µm × 10 µm. Before freezing in liquid nitrogen for further diffraction data collection, the crystals were transferred into the well solution as cryosolution.

Crystals of the anti-IL-17A-N were obtained by the vapor-diffusion method using as precipitant solution No. 21 of JCSG-plus Screen I (Molecular Dimensions), containing 0.1 M Citrate pH 5, 20% (*w*/*v*) PEG 6000. Drops were made by mixing protein solution at 45 mg mL^−1^ in 50 mM Na–Ac pH 5.5, 150 mM NaCl with well solution in a 1:1 volume ratio. Crystals appeared after 45 days and grew to maximum dimensions of 220 µm × 40 µm × 200 µm. Before freezing in liquid nitrogen for further diffraction data collection, the crystals were transferred into the well solution as cryosolution.

### 4.6. Diffraction Data Collection and Determining the IL-17A/Anti-IL-17A-76 Complex and Anti-IL-17A-N Mutant Form Structures

The diffraction data were collected on ID30A-3 beamline at the ESRF electron storage ring (Grenoble, France) and processed and merged using the XDS package. The crystal structure of anti-IL-17A-76 bound to IL-17A was solved by molecular replacement method with Phaser using deposited structures of IL-17A (PDB code 4HSA) and V_H_H domain of netakimab Fab fragment (PDB code 6QKD) as the start models. The initial model was subjected to crystallographic refinement with REFMAC5 [35]. Manual rebuilding of the model was carried out in Coot [36]. Data and refinement statistics are summarized in Table 6. The coordinates and structure factors of IL-17A/anti-IL-17A-76 complex and anti-IL-17A-N mutant form crystal structures have been deposited in the Protein Data Bank (PDB code 8B7W, PDB code 6RBB). Figures were prepared using PyMOL [37].

## 5. Conclusions

Thus, the structural analysis of interactions in IL-17A/antibody complexes showed that the solvent-inaccessible H-bonds formed by IL-17A β-turn residues are crucial to epitope–paratope interactions. We assume that the dominant role in the IL-17A/V_H_H domain complex belongs to the epitope-1 region located in the β-turn of IL-17A. Further stabilization of the complex is provided through the contacts of the mobile IL-17A C-terminal residues (epitope-2) with the CDR-H3 loop of the V_H_H domain.

## Figures and Tables

**Table 1:** Kinetic analysis of interactions of IL-17A with V_H_H domain modifications.

	K_on_ (M^−1^ s^−1^)	K_off_ (s^−1^)	K_D_ (nM)
anti-IL-17A	5.0 × 10^5^	4.3 × 10^−5^	0.09
anti-IL-17A-76	4.7 × 10^5^	2.2 × 10^−5^	0.06

**Table 2:** Kinetic analysis of interactions of IL-17A with netakimab antibody and its fragments—the Fab fragment and the V_H_H domain [26].

	K_on_ (M^−1^ s^−1^)	K_off_ (s^−1^)	K_D_ (nM)
Netakimab	2.0 × 10^5^	1.0 × 10^−7^	<0.001
Fab fragment	6.3 × 10^4^	2.0 × 10^−5^	0.3
V_H_H domain	5.0 × 10^5^	4.3 × 10^−5^	0.09

**Table 3:** Anti-IL-17A and anti-IL-17A-N amino acid sequence alignment in the N-terminal region. Amino acids of the altered region are shown in italic, matching amino acids are in bold.

Number of Amino Acid	1 11
Anti-IL-17A	* QVQLVQSGGG* **LVQA**
Anti-IL-17A-N	* RGSEFEVQ*-------- **LVQA**

**Table 4:** Kinetic constants of the interactions of IL-17A with anti-IL-17A mutant forms.

	K_on_ (M^−1^ s^−1^)	K_off_ (s^−1^)	K_D_ (nM)
anti-IL-17A-76	4.7 × 10^5^	2.2 × 10^−5^	0.06
anti-IL-17A-N	4.8 × 10^5^	2.5 × 10^−5^	0.05

**Table 5:** IL-17A-specific antibodies. K_D_ for cytokine/antibody complexes and half-maximal inhibitory concentrations are listed.

	K_D_ (nM)	IC_50_ (pM)	Reference
netakimab Fab fragment	0.30	66	[29]
netakimab V_H_H domain	0.09	30	[24]
secukinumab	0.12	201	[30]
CAT-2200 Fab fragment	2.10	1560	[31]
HB0017	0.01	56	[32]

**Table 6:** Data collection and refinement statistics.

Data Collection		
	IL-17A/Anti-IL-17A-76 Complex	Mutant Form Anti-IL-17A-N
Space group	P3_1_2_1_	P22_1_2_1_
a, b, c, (Å)	71.05, 106.04	54.87, 72.49, 129.38
α, β, γ, (°)	90.0, 120.0, 90	90, 90, 90
Resolution limits, Å	50.0–2.85 (2.92–2.85)	50.0–2.38 (2.52–2.38)
R_sigma_, %	25.3 (182.9)	13.8 (116.9)
Mean I/σ(I)	12.00 (1.58)	9.10 (1.40)
Completeness, %	98.1 (99.4)	98.2 (90.7)
Redundancy	6.73 (7.11)	5.18 (5.06)
CC_1/2_ (%)	95.9 (46.2)	99.6 (80.8)
Unique reflections	7466 (538)	21,024 (3 076)
Refinement statistics		
Resolution, Å	40.17–2.85 (3.26–2.85)	41.85–2.45 (2.51–2.45)
Total number of reflections	7460 (2336)	19,563 (2702)
R_work_/R_free_, %	22.2/26.8 (23.9/31.8)	21.5/28.01 (33.75/38.07)
Average B-factor, Å^2^	71.0	41/0
Ramachandran plot		
Most favorable regions, %	94.2	96.84
Allowed regions, %	5.8	3.16
R.m.s. deviations		
Bond length, Å	0.009	0.009
Bond angle, °	1.109	1.053

Note: Values in parentheses are for the last resolution shell.

## Data Availability

PDB code 8B7W.

## Supplementary Materials

The following supporting information can be downloaded at: https://www.mdpi.com/article/10.3390/ijms232314904/s1.
