# Supplementary material for: Two Epitope Regions Revealed in the Complex of IL-17A and Anti-IL-17A VHH Domain
Source: Int J Mol Sci. 2022 Nov 28;23(23):14904. doi: 10.3390/ijms232314904 (PMC9738047; doi:10.3390/ijms232314904)
Supplement: Supplementary file 1 [file ijms-23-14904-s001.zip › ijms-1993246-supplementary.pdf]

International Journal of *Molecular Sciences*

MDPI

Table S1. The main interface of complex IL-17A/anti-IL-17A-76:

The list of contacts of IL-17A with antibody in the complexes with V_HH (8B7W), Secukinumab (PDB 6WIO), and HB0017 antibody (PDB 7WKX).

	**IL-17A/<br>anti-IL-17A-76 (8B7W)**	**IL-17A/IL-17RA<br>(4HSA)**	**IL-17A/secukinumab (6WIO)**		**IL-17A/HB0017 (7WKX)**	
	anti-IL-17A-76		H-chain	L-chain	H-chain	L-chain
Contact surface area, Å^2	840+120<br>**960**	1228+993<br>**2221**	423+241<br>**664**	246+171<br>**417**	540+99<br>**639**	263
H-bonds of antibody with the molecule A of IL-17A	7<br>Tyr108OH-Asp56OD1<br>Leu97N-Asp56OD2<br>Asn111ND2-Gly100O<br>Asn111OD1-Arg95NH2<br>Ile150N-Asp99O<br>Val151N-Asp99O<br>Cys94O-Ser53N	15<br>Ser64O- Asn89ND2<br>Asp65N-Asp123O<br>Ser63OG-Gln124OE1<br>Asp65OD1-Glu125N<br>Pro82O-Trp31NE1<br>Arg124NE-Trp31O<br>Arg124NH2-Trp31O<br>Val88O-Arg93NH2<br>Trp90N-Asn91OD1<br>Trp90O-Asn91ND2<br>Asn55ND2-Glu92OE2<br>Arg69NH2-Asp262OD1<br>Arg69NH1-Asp262OD2<br>Arg78NH1-Glu127OE1<br>Arg124NH2-Pro136O	5<br>Asn111ND2-Lys58O<br>Asn111OD1-Lys58N<br>Val106O-Lys65NZ<br>Tyr108O-Tyr60N<br>Pro149O-His110ND1	3<br>His153NE2-Tyr33OH<br>His152NE2-Gly93O<br>His109O-Ser95OG	6<br>Ser87OG-Asn31O<br>Asn53N-Asn54O<br>Arg124NH1-Tyr103OH<br>Glu80OE1-Tyr32OH<br>Pro82O-Asn31ND2<br>Asn53ND2-Thr55O	3<br>Asp81OD1-Asn33ND2<br>Glu83OE2-Tyr37OH<br>Glu83OE2-Lys55NZ
H-bonds of antibody with	1<br>Tyr67OH-Asp99OD1	14<br>Ser112O-Asn261ND2	4<br>Arg69NH1-Glu57OE1	4<br>Ala102O-Ser95N	0	0

the molecule A* of IL-17A		Leu97O-Asn261ND2<br>Asn111ND2-Asn261O<br>Asn111ND2-Asn262OD1<br>Tyr108O-Arg265NH1<br>Asn111OD1-Arg265NH2<br>Tyr108O-Arg265NH2<br>Ile150O-Arg265N<br>His152N-Arg265O<br>His152O-Ser267N<br>Val154N-Ser267O<br>Glu118OE1-Arg93N<br>His109NE2-Thr204OG1<br>Gln117NE2-Asn89O	Arg69NH2-Glu57OE2<br>Tyr66OH-Ser56OG-through the water molecule<br>Arg62NE-Asp106O	Asp103OD1-Ser94OG<br>Asp103O-Ser30OG<br>Asp103OD2-Gln27NE2		
Salt bridges	0	0	0	0	0	0
H-bonds inaccessible to the solvent on both IL-17A molecules	6<br>Leu97N-Asp56OD2<br>Asn11ND2-Gly100O<br>Asn11OD1-Arg95NH2<br>Ile150N-Asp99O<br>Val151N-Asp99O<br>Cys94O-Ser53N	6<br>Trp90N-Asn91OD1<br>Asn111ND2-Asn261O<br>Ile150O-Arg265N<br>His152N-Arg265O<br>His152O-Ser267N<br>Val154N-Ser267O	4<br>Asn111ND2-Lys58O<br>Asn111OD1-Lys58N<br>Tyr108O-Tyr60N<br>Pro149O-His110ND1	1<br>Ala102O-Ser95N	1<br>Pro82O-Asn31ND2	1<br>Glu83OE2-Tyr37OH
Hydrophobic interactions on antibody or receptor	Ala30<br>Thr31<br>Pro33<br>Ala50<br>Ile51<br>Pro52A<br>Ile58<br>Arg97<br>Phe98<br>Gly100	Thr25<br>Cys26<br>Leu27<br>Trp31<br>Ile32<br>Pro34<br>Leu86<br>Leu88<br>Pro138<br>Ser258	Trp 33<br>Trp47<br>Ala50<br>Glu57<br>Tyr59<br>Tyr60<br>Gly62<br>Lys65<br>Ile109<br>Trp112	Pro96<br>Ser95<br>Ser94<br>Gly93	Tyr27<br>Phe29<br>Asn31<br>Tyr32<br>Trp50<br>Trp101<br>Gly102	Val99

	Tyr100C<br>Tyr100D	------------<br>His33<br>Thr90<br>Ile176<br>Leu202<br>Phe255<br>Leu264<br>Arg265<br>His266<br>Ala268				
Hydrophobic interactions on the molecule A of IL-17A	His77<br>Glu91<br>Lys93<br>Cys94<br>Leu97<br>Tyr108<br>Val113<br>Pro114<br>Ser141<br>Val147<br>Pro149<br>Ile150<br>Val151<br>His153	Leu49<br>Ile51<br>Pro60<br>Lys61<br>Leu76<br>Arg84<br>Tyr85<br>Pro86<br>Val88<br>Trp90<br>Arg124<br>Pro127<br>Phe133	Leu97<br>Ty108<br>His109<br>Met110 main chain<br>Asn111<br>Val113<br>Val 147<br>Thr148 main chain<br>Pro149<br>Ile150<br>Val151	His109<br>Thr148<br>Ile150	Pro82<br>Glu83<br>Arg84<br>Tyr85<br>Ser87<br>Val88<br>Phe133	Pro82<br>Pro127
Hydrophobic interactions on the molecule A* of IL-17A	Tyr66<br>Arg62	Arg43<br>Val45<br>Leu97<br>Tyr108<br>His109<br>Val113<br>Leu139	Arg62	-	Arg43<br>Val45<br>Leu135	-

	Pro149	
	Ile150	
	Val151	
	His152	
	His153	
	Val154	

Table S2. Crystal contact of complex IL-17A/anti-IL-17A-76.

Contact surface area of anti-IL-17A-76 with the molecule A of IL-17A, Å^2	**577**
H-bonds of antibody with the molecule A of IL-17A	Arg69NH2-Gln6O<br>Asp65OD2-Gly8N<br>Asp68OD2-Leu108N<br>Arg78NH2-Ser112OG<br>Arg78NH2-Ser113O<br>Arg78NE-Ser112OG<br>Asp68O-Gln105NE2<br>Ser70O-Gln105NE2
Hydrophobic interactions on anti-IL-17A-76 with the molecule A of IL-17A	Leu11<br>Val89<br>Leu108<br>Thr110
Hydrophobic interactions on the molecule A of IL-17A	Leu76<br>Trp90<br>Arg95<br>Asn75
Contact surface area of anti-IL-17A-76 with the molecule A* of IL-17A, Å^2	85
H-bonds of antibody with the molecule A* of IL-17A	0
Hydrophobic interactions on anti-IL-17A-76 with the molecule A* of IL-17A	Val5
Hydrophobic interactions on the molecule A* of IL-17A	Ile150

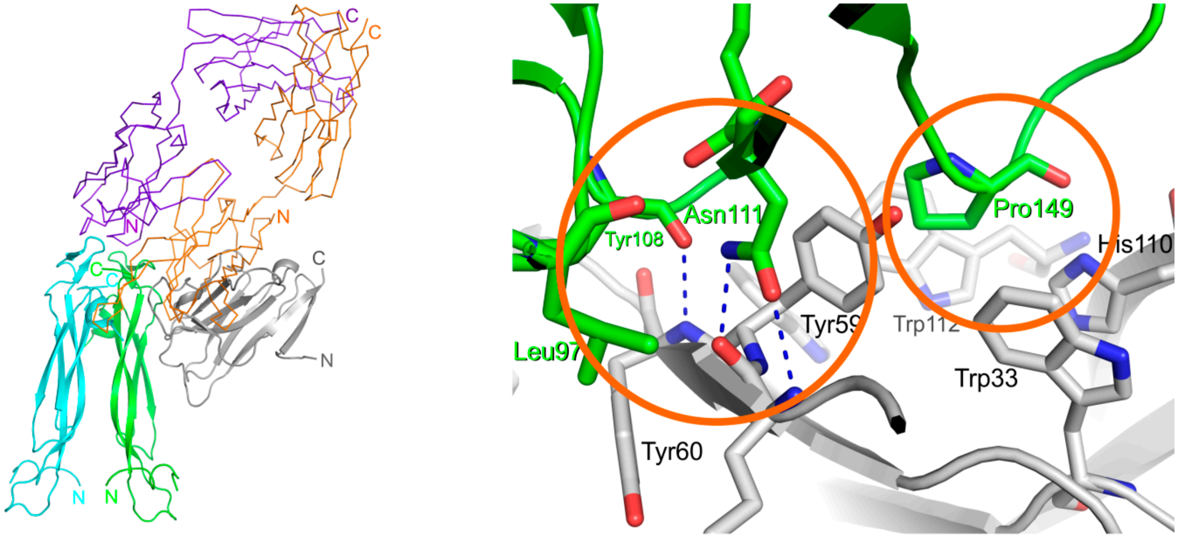

(a) (b)

Figure S1

a. Structure overlay of IL-17A with the secukinumab Fab fragment, PDB code 6WIO (heavy chain is shown in purple, light chain in yellow) and with the anti-IL-17A-76, PDB code 8B7W (grey). IL-17A monomers are shown in blue and green.

b. The epitope-paratope region in the IL-17A/secukinumab Fab fragment complex. The hydrogen bonds between Asn 111 and Tyr 108 (the epitope-1) and the Fab fragment heavy chain CDR region are shown. The epitope-2 includes contacts between Pro 149, Ile150, and Val 151 of IL-17A with the antibody. IL-17A is shown in green, secukinumab is shown in grey. Ellipses highlight two areas of the epitope.

```
        10         20         30         40
MTPGKTSLVS LLLLLSLEAI VKAGITIPRN PGCPNSEDKN
          signal peptide

        50         60         70         80
FPRTVMVNLN IHNRNTNTNP KRSSDYYNRS TSPWNLHRNE

        90        100        110        120
DPERYPSVIW EAKCRHLGCI NADGNVDYHM NSVPIQQEIL

       130        140        150
VLRREPPHCP NSFRLEKILV SVGCTCVTPI VHHVA   155
```

(a)

```
        10         20         30         40
MTPGKTSLVS LLLLLSLEAI VKAGITIPRN PGCPNSEDKN
          signal peptide

        50         60         70         80
FPRTVMVNLN IHNRNTNTNP KRSSDYYNRS TSPWNLHRNE

        90        100        110        120
DPERYPSVIW EAKCRHLGCI NADGNVDYHM NSVPIQQEIL

       130        140        150
VLRREPPHCP NSFRLEKILV SVGCTCVTPI VHHVA   155
```

(b)

Figure S2. Amino acid sequence of IL-17A. The signal peptide amino acids are shown in blue. a. Contacts of anti-IL-17A-76 with the IL-17A molecule A are shown in green, with the molecule A* in pink. **b.** H-chain contacts of secukinumab with the IL-17A molecule A are shown in light green, with the molecule A* in pink. L-chain contacts of secukinumab with the IL-17A molecule A are shown in dark green, with the molecule A* in red. The crossing contacts of secukinumab and anti-IL-17A-76 with IL-17A are boxed.

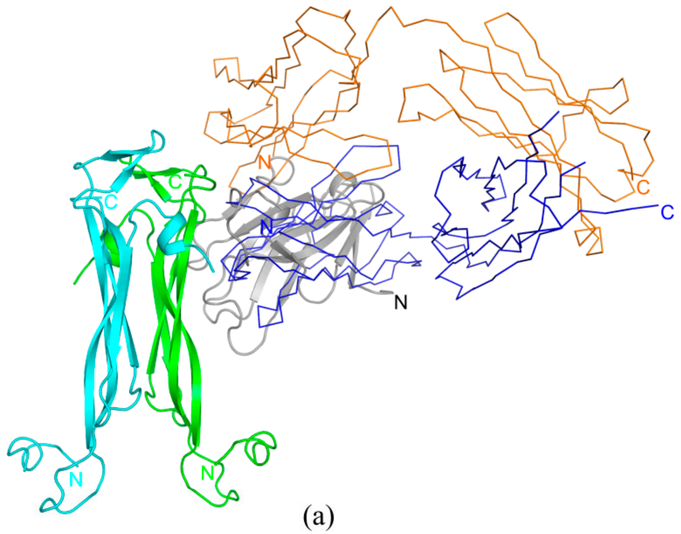

(a)

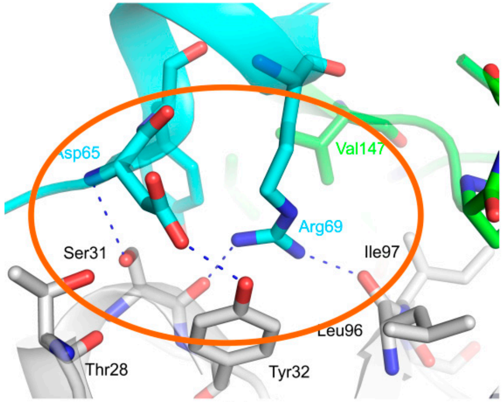

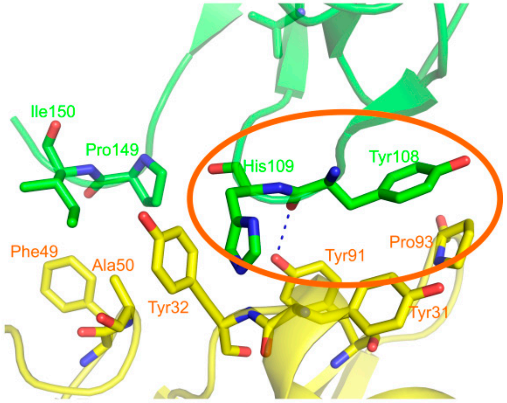

(b) (c)

Figure S3 a. Superposition of structures of IL-17A complexed with the CAT-2200 Fab fragment, PDB code 2VXS (heavy chain is shown in dark blue, light chain in yellow, IL-17A monomers are shown in cyan and green) and with the anti-IL-17A-76, PDB code 8B7W (grey). The epitope regions located on molecules A **(b)** and A* **(c)** of IL-17A (amino acids of IL-17A are shown in blue and green) in the complex with the CAT-2200 Fab fragment (the heavy chain is shown in grey and the light chain in yellow). Ellipses highlight two areas of the epitope.

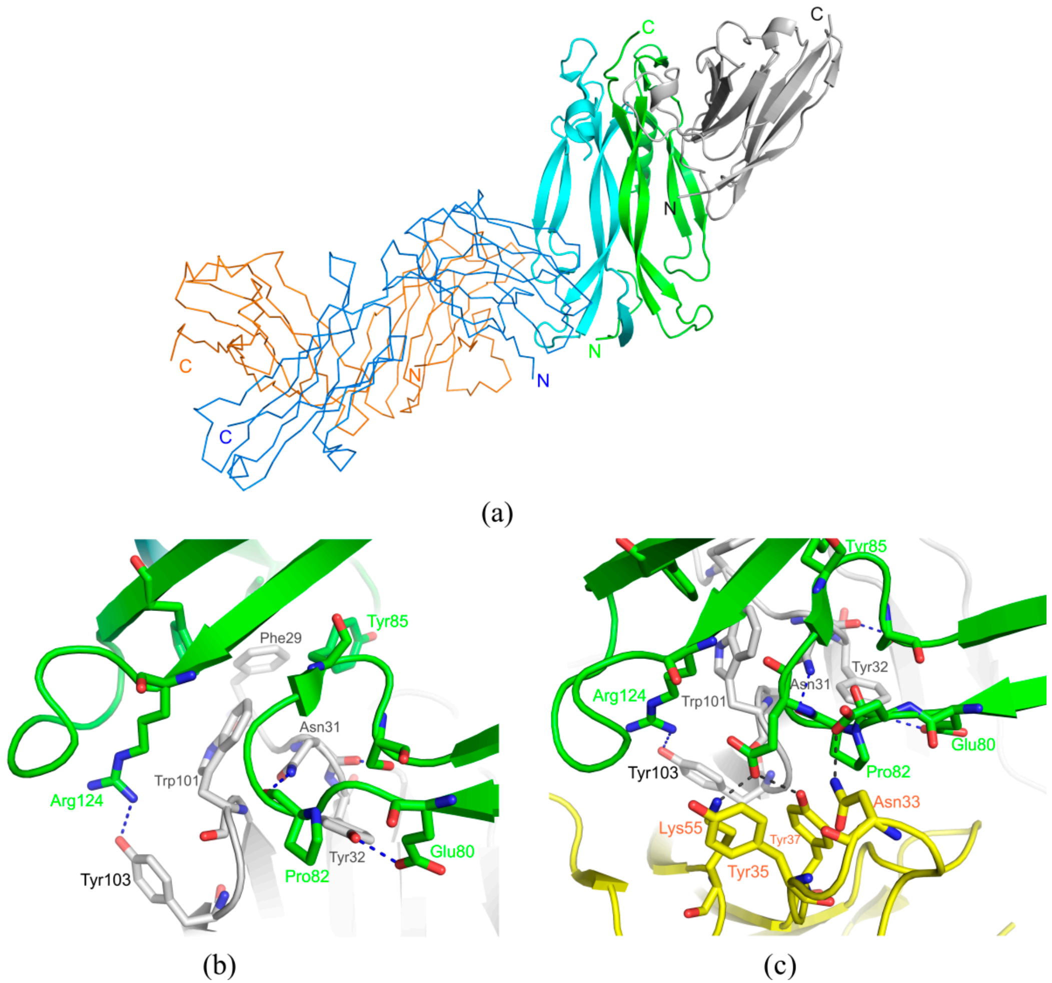

Figure S4. a. Superposition of structures of IL-17A complexed with the HB0017 Fab fragment (PDB code 7WKX, the heavy chain is shown in blue and the light chain in yellow) and the anti-IL-17A-76 (PDB code 8B7W, grey). IL-17A monomers are shown in cyan and green. The epitope-paratope area of IL-17A (green) and: **b.** Contacts of IL-17A with the HB0017 heavy chain antibody (grey). **c.** Contacts of IL-17A with the HB0017 light chain (yellow).
